# Low-Cost and Rapid Fabrication of Metallic Nanostructures for Sensitive Biosensors Using Hot-Embossing and Dielectric-Heating Nanoimprint Methods

**DOI:** 10.3390/s17071548

**Published:** 2017-07-02

**Authors:** Kuang-Li Lee, Tsung-Yeh Wu, Hsuan-Yeh Hsu, Sen-Yeu Yang, Pei-Kuen Wei

**Affiliations:** 1 Research Center for Applied Sciences, Academia Sinica, Taipei 11529, Taiwan; 2 Department of Mechanical Engineering, National Taiwan University, Taipei 10617, Taiwan; d97522026@ntu.edu.tw (T.W.); syyang@ntu.edu.tw (S.Y.); 3 Institute of Optoelectronic Sciences, National Taiwan Ocean University, Keelung 20224, Taiwan; adamsyu714@gmail.com

**Keywords:** metallic nanostructures, biosensors, Fano resonance, hot-embossing, radio-frequency heating, template-stripping

## Abstract

We propose two approaches—hot-embossing and dielectric-heating nanoimprinting methods—for low-cost and rapid fabrication of periodic nanostructures. Each nanofabrication process for the imprinted plastic nanostructures is completed within several seconds without the use of release agents and epoxy. Low-cost, large-area, and highly sensitive aluminum nanostructures on A4 size plastic films are fabricated by evaporating aluminum film on hot-embossing nanostructures. The narrowest bandwidth of the Fano resonance is only 2.7 nm in the visible light region. The periodic aluminum nanostructure achieves a figure of merit of 150, and an intensity sensitivity of 29,345%/RIU (refractive index unit). The rapid fabrication is also achieved by using radio-frequency (RF) sensitive plastic films and a commercial RF welding machine. The dielectric-heating, using RF power, takes advantage of the rapid heating/cooling process and lower electric power consumption. The fabricated capped aluminum nanoslit array has a 5 nm Fano linewidth and 490.46 nm/RIU wavelength sensitivity. The biosensing capabilities of the metallic nanostructures are further verified by measuring antigen–antibody interactions using bovine serum albumin (BSA) and anti-BSA. These rapid and high-throughput fabrication methods can benefit low-cost, highly sensitive biosensors and other sensing applications.

## 1. Introduction

Surface plasmon resonance (SPR) is a label-free optical technique for real-time detection of biomolecular interactions and can be utilized for many applications, such as drug development, disease diagnostics and environmental monitoring [1,2,3,4]. Commercial SPR methods use an optical prism for coupling incident polarized light into surface plasmon polariton (SPP) on a gold thin film. Different from the prism-coupling method, metallic nanostructures can directly excite SPP using normal incidence without any prism. The metallic nanostructures have been applied for various kinds of sensors [5,6,7,8,9,10,11,12]. They possess many benefits, including small detection volume, simple measurement setup, and ease of multiple detections. To evaluate the biosensing capability of metallic nanostructures with intensity interrogation, a figure of merit for biolayer thickness (FOM_t_) is utilized [13,14]. The FOM_t_ is defined as

(1) $$FOM_{t}=(\frac{S_{\lambda }(n_{a}-n_{s})}{l_{d}}){\left(\frac{\left(\Delta I/\Delta \lambda \right)}{I}\right)}_{\max}$$

where *S_λ_* is the bulk wavelength sensitivity (wavelength shift/refractive index change), *ΔI/I* is the intensity sensitivity at a certain wavelength, *n_s_* is the bulk solution refractive index, *n_a_* is the adsorbate monolayer refractive index, and *l_d_* is the evanescent length of SPP. The biolayer sensitivity is determined by wavelength sensitivity, evanescent length, and refractive index difference between the adsorbate monolayer and surrounding environment, and the intensity sensitivity. To improve the sensing capability of SPR sensors, many methods have been proposed, such as spectral integration analysis [15,16,17,18], thermal-annealing nanoimprint method [19,20,21,22], Fano coupling method [23,24,25], narrowing resonance bandwidth with oblique angle incidence [26], two-mode coupling between top and substrate resonances [27], oblique-angle-induced Fano resonances [13], and nearly guided wave SPR sensors [28]. Among these approaches, the Fano resonance is frequently utilized to increase the intensity sensitivity in metallic nanostructures. There is spectral overlapping between broadband resonance and narrowband resonance in the Fano resonance [24]. The broadband spectrum comes from incident light or cavity resonance in metallic nanostructures. The narrowband resonance is attributed to the SPR mode or localized SPR mode. Such spectral overlapping results in an asymmetric sharp resonant profile, and thus increases the intensity sensitivity. Fano resonances have been found in multiple nanoparticles [5], periodic metallic nanostructures [9,25], and metamaterials [29]. However, mass production of Fano-resonance structures with low-cost and high-throughput is important for commercial applications. Conventional nanofabrication techniques use focused ion beam (FIB) to mill metal films, or electron-beam (EB) lithography to make nanostructures on the EB resist. These nanofabrication techniques are expensive, and cannot be used for mass production. Many methods have been proposed to solve the mass production problem, such as optical interference lithography [30], thermal or UV nanoimprint lithography (NIL) [15,31], nanosphere lithography [32], nanostencil lithography [33], and the template-stripping method [20,21,34,35]. Typically, the template-stripping method uses a patterned silicon template which is coated with a gold thin film. Due to the poor adhesion of gold film to the silicon, the gold film with patterned structures is transferred to the substrate with an epoxy adhesive. In our previous work [22], we proposed to make gold nanostructures on plastic substrates by using the thermal-annealing template-stripping method. In contrast to the use of epoxy adhesive, nanostructures were directly made on a plastic surface using a thermal-annealing approach, with a temperature higher than the glass-transition temperature (T_g_) of the plastic substrate. The template-stripping method does not require additional photoresist, etching or lift-off processes. After peeling off from the silicon template, the gold periodic nanostructure achieved ~10 nm SPR spectral width.

However, the thermal-annealing template-stripping method requires removal of residues on the template after each imprinting process, which makes it difficult for massive fabrication. Besides, the method is only suitable for gold nanostructures, due to poor adhesion of gold film to the silicon surface. In our experience, silver and aluminum nanostructures cannot be well transferred to plastic films. To solve the problem for massive and rapid fabrication with different metallic nanostructures, we developed two methods—rapid hot-embossing and dielectric-heating nanoimprinting techniques—for rapid and low-cost fabrication of nanostructures. Each imprinting process was completed within several seconds, and no release agent or epoxy was required. We utilized the hot-embossing nanoimprinting method and thermal evaporation to fabricate low-cost, large-area, and highly sensitive aluminum nanostructures on A4 size plastic films. The transmission spectrum in a 470 nm-period capped aluminum nanoslit array showed a Fano resonance with a bandwidth only 2.7 nm. As the proposed nanostructure has an extremely sharp resonance in the visible light region, it achieves a figure of merit (FOM) of 150, and an intensity sensitivity of 29,345%/RIU (refractive index unit). In the dielectric-heating method, we used a radio-frequency (RF) sensitive polymer film, polyethylene terephthalate glycol-modified (PETG), combined with a commercial RF-welding machine for the rapid fabrication of various nanostructures. The capped aluminum nanoslit arrays achieved a linewidth of 5 nm and bulk refractive index sensitivity of 490.6 nm/RIU. In addition, the sensing capabilities of the aluminum nanostructures were verified by measuring bovine serum albumin (BSA) and anti-BSA interactions. Such low-cost, rapid, and high-throughput fabrication of highly sensitive SPR sensors can benefit commercial applications.

## 2. Materials and Methods

### 2.1. Rapid Hot-Embossing Nanoimprinting Process for Metallic Nanostructures

**Figure 1:**
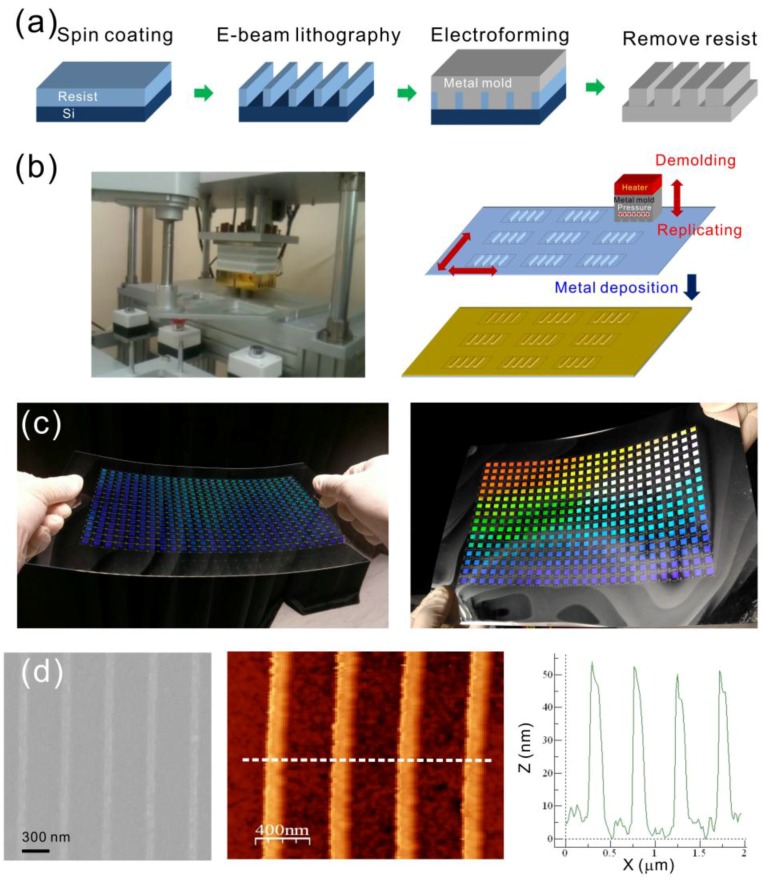
(**a**) The fabrication process for Ni–Co mold; (**b**) the homemade hot-embossing machine for rapidly generating arrays of nanostructures on an A4 size plastic film; (**c**) the optical image of the replicated nanostructure (left) and capped aluminum nanoslit arrays (right) on an A4 size polycarbonate film. There are 416 arrays and the area of each periodic nanostructure is 5 × 5 mm^2^; (**d**) the SEM (left), AFM (middle), and cross-sectional profile (right) of the capped aluminum nanoslits.

Figure 1a shows the fabrication process for the metal mold. The nanostructures were produced on a polycarbonate (PC) film using a rapid hot-embossing approach. For the fabrication of the mold, a 100 nm-thick diluted ZEP-520 resist (ZEP-520, Zeon Corp, Tokyo, Japan) was spin-coated onto a 525 μm-thick silicon substrate. Periodic nano-grooves of 60 nm width, 100 nm depth, and 470 nm period, were fabricated in a resist using an EB writing system (ELS-F125, Elionix, Japan). The patterns were then coated with gold and electroformed with Ni and Co to produce a 250 μm-thick metal mold. For the rapid hot-embossing method, the Ni–Co mold is important. It has low adhesion to plastic films, and can stand hitting force during the rapid stamping process. The Ni–Co mold was heated at a temperature between 170 °C and 190 °C. This temperature is much higher than the T_g_ of the PC (135 ^o^C), in order to rapidly heat the surface of the plastic film. The embossing pressure was 140 psi. The nanostructures on the mold were imprinted on a 178 µm-thick PC film using a homemade hot-embossing machine, as shown in Figure 1b. After imprinting the nanostructure onto the plastic film, the Ni–Co mold was directly separated from the replicated plastic film without cooling the temperature of the mold. The time needed for a single stamping process was only 5 s. The nanostructures can be rapidly replicated, and the Ni–Co mold can be used repeatedly. With different heating temperatures on the Ni–Co mold, the nanostructures with different ridge heightes, from 35 to 75 nm, were made. After depositing an aluminum film with a thickness of 42 nm on the imprinted plastic substrates, the capped aluminum nanoslit arrays were produced. Figure 1c show optical images of the replicated nanostructure arrays on an A4 size PC film, and the capped aluminum nanoslit arrays on an A4 size PC film, respectively. There are 416 arrays, and the area of each periodic nanostructure is 5 × 5 mm^2^. Figure 1d shows the SEM, AFM and cross-sectional images of the capped aluminum nanoslits with a ridge height of 50 nm. This machine can be used to fabricate arrays of metallic nanostructures for high-throughput detections.

### 2.2. RF-Heating Nanoprinting Process for Metallic Nanostructures

**Figure 2:**
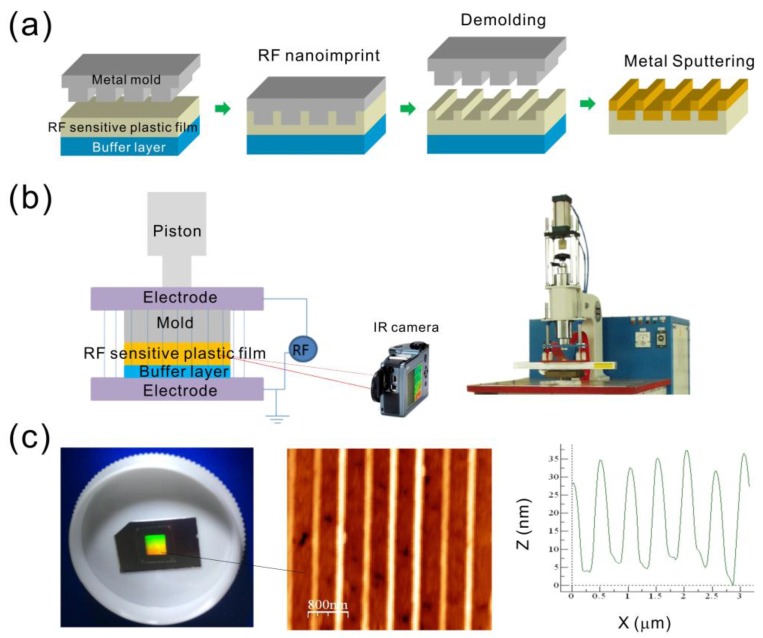
(**a**) The fabrication process for the capped metallic nanoslits using the RF-heating nanoimprinting method and metal coating; (**b**) setup of the radio-frequency (RF)-heating method. The commercial RF machine is shown in the inset; (**c**) the optical (left), AFM (middle), and cross-sectional profile (right) of the capped aluminum nanoslits. The RF power: 2.17 KW, RF time: 9 s, pressure: 106 psi.

Figure 2a shows the fabrication process for fabricating nanostructures using the dielectric-heating nanoimprinting method, which consists of three steps: dielectric-heating, molding/demolding and sputtering of a metallic thin film. Dielectric-heating is an electronic heating process using a high-frequency alternating electric field to heat a dielectric material. In our method, we used a commercial RF welding machine (HG-501S, Hexagon Electric Industrial Co., Taiwan) operated at 27.12 MHz. The RF-heating system is shown in Figure 2b. At this RF frequency, the molecular dipole rotation within certain polymers will cause very rapid heating. The widely used polymers for RF heating include PVC (polyvinylchloride), polyamides (PA), nylon, PETG and some ABS (acrylonitrile butadiene styrene) plastics. In our experiment, we used PETG (T_g_ ~80 °C) as the RF-sensitive polymer. Compared to thermal heating, RF heating requires a moderate amount of energy. The fabrication parameters are RF power, RF-active time, and pressure. To reduce the heating time, the mold was preheated to a temperature below the T_g_ of the polymer. The preheating temperature was set at approximately 60 °C, and the heating power was 3.5 kW. The polymer film reached a temperature higher than the T_g_. Applying pressure on the mold embossed the nanopatterns onto the softened polymer film for several seconds. The temperature of RF-sensitive polymers was monitored using an infrared thermometer. It increased approximately 30 °C within 9 s. This indicates that RF can induce molecular vibrations, and generate heat in RF-sensitive polymers in a short time. The temperature in the RF-sensitive polymer rapidly decreased when the RF power was turned off. The mold and the polymer substrate can be easily separated soon after dielectric-heating. The nanostructures can be rapidly replicated, and the mold can be used repeatedly. After evaporating a metallic film with a thickness of 50 nm on the imprinted polymer substrates, the capped metallic nanoslit arrays were produced. Figure 2c shows the optical, AFM and cross-sectional images of the capped aluminum nanoslits with a ridge height of 30 nm.

### 2.3. Transmission Spectrum Measurement of Metallic Nanostructures

A white light source (60 W lamp) was coupled to a fiber cable, with the output mounted with a fiber lens for collimating incident light. The incident light passed through a linear polarizer to form a transverse-magnetic (TM) polarized light, and was focused on the metallic nanostructures. The transmission light from the metallic nanostructures was collected by another fiber lens and focused on a fiber cable. The transmission spectra were measured using a fiber coupled spectrometer (BWTEK, BTC112E).

### 2.4. Refractive Index Sensitivity Tests and Biosensing Experiments

The bulk wavelength sensitivity (*S_λ_*) of metallic nanostructures was examined using media with different refractive indexes. The media were prepared using water mixed with various fractions of glycerin. The refractive index of the media ranged from 1.333 to 1.355. The sensitivity was determined from the change of resonant wavelength with refractive index change. The biosensing experiments were tested by using bovine serum albumin (BSA; Sigma-Aldrich) and anti-BSA (Sigma-Aldrich) in a deionized (DI) water buffer. To immobilize BSA on the nanostructure surface, the SPR chips were first exposed to a 10% aminopropyltriethoxysilane (APTES) solution for 30 min, and baked at 120 °C for one hour. This process will form amino groups on the chip surface. After surface modification, 100 μL solution of 1 mg/mL BSA was put onto the chip. After one hour of incubation, the chip was washed with DI water in order to remove unbound BSA molecules. The SPR chip was then dried by blowing with nitrogen gas. For the antigen–antibody interactions, 100 μL solution of anti-BSA was put on the structure surface for one hour. The chip was then washed with DI water, and blown dry by nitrogen gas. These processes were subsequently repeated for different concentrations of anti-BSA solutions from 100 pg/mL to 1 mg/mL. The transmission spectrum measurements were conducted before and after BSA and anti-BSA interactions.

## 3. Results

### 3.1. Optical Properties of Metallic Nanostructures Fabricated by Rapid Nanoimprinting Method

**Figure 3:**
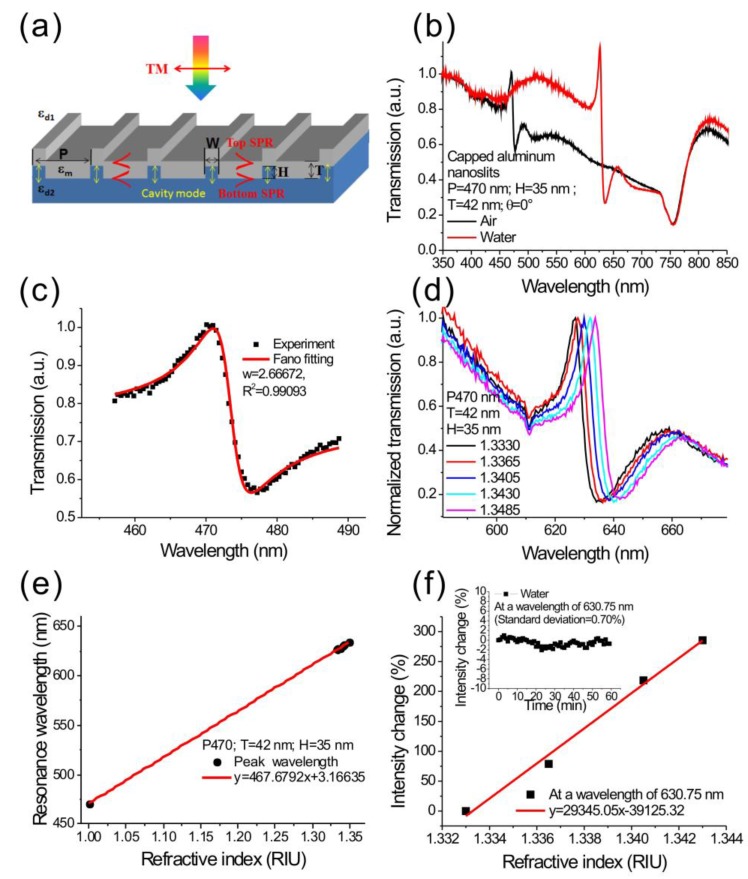
(**a**) The structure parameters of the capped aluminum nanoslits and the direction of the polarized incident light; (**b**) measured transmission spectra of the periodic capped aluminum nanoslits made by rapid hot-embossing method; (**c**) the Breit–Wigner–Fano fitting of the Fano resonance; (**d**) the transmission spectra of the capped aluminum nanoslits with various water/glycerin mixtures; (**e**) the peak wavelength shift against the refractive index of the medium for the nanostructure. The wavelength sensitivity was 467 nm/RIU; (**f**) the normalized intensity change against the refractive index. The intensity sensitivity was 29,345%/RIU. The inset shows the intensity noise as a function of time.

Figure 3a shows the geometrical parameters of capped aluminum nanoslits and the polarization direction of incident light. Figure 3b shows the measured transmission spectra in air and water for normally incident TM-polarized light. The parameters for capped aluminum nanoslits were H = 35 nm, T = 42 nm, W = 60 nm, and P = 470 nm. The sample was made using the rapid hot-embossing method. A clear Fano resonance was found in the spectrum. The Fano resonance comes from the couplings between cavity resonance within metallic nanoslits, and SPR modes on the periodic metallic surface (the aluminum/medium and aluminum/substrate interfaces). The gap plasmons within the nanoslits had a broadband transmission due to the Fabry–Perot effect [36],

(2) $$2n_{eff}k_{0}h+\phi_{1}+\phi_{2}=2m\pi ,$$

where *h* is the height of the periodic ridge, *k*_0_ is the free space wavelength vector *(2π/*λ_0_), *n_eff_* is the equivalent refractive index in the slit, and *ϕ*_1_ and *ϕ*_2_ are the phase shifts at the top and bottom interfaces. The cavity resonance wavelength can be estimated by the slit width and height. The measured cavity resonance wavelength in water was 515 nm. The SPR mode comes from the Bloch wave SPP on a periodic metallic surface. It occurs when the Bragg wave condition is satisfied. The Bragg condition for one-dimensional arrays can be described by [1]

(3) λSPR(n,i)=Pi{Re[(εmn2εm+n2)1/2]},

where *i* is the resonance order, *P* is the period of the nanostructure, *ε_m_* is the relative permittivity of the metal, and *n* is the environmental refractive index. The measured resonance wavelengths of Fano resonances at the air/aluminum and substrate/aluminum interfaces were 477 and 755 nm, respectively. Such resonances can be fitted by the Fano resonance equation (Breit–Wigner–Fano line shape),

(4) $$T_{Fano}(\lambda )=T_{a}+\frac{T_{b}{\left(1+\frac{\lambda -\lambda_{c}}{qw}\right)}^{2}}{1+{\left(\frac{\lambda -\lambda_{c}}{w}\right)}^{2}},$$

where *T_a_*, *λ_c_*, *T_b_*, and *w* are the slowly varying transmittance, resonance wavelength, contribution of broadband light that couples with the narrowband resonance, and linewidth of the resonance, respectively. *q* is the Breit–Wigner–Fano parameter which describes the coupling strength. According to the fitting equation, the Fano resonance at the air/aluminum interface, the linewidth (*w*) and Fano factor (*q*) was 2.7 nm and −1.13 as seen in Figure 3c. To the best of our knowledge, it is the narrowest bandwidth observed in aluminum nanostructures. When the array was covered with water, the Fano resonance at the air/aluminum interface was redshifted to 635 nm wavelength, and the resonance at the substrate/aluminum interface remained unchanged. It was noted that a similar resonance profile is reported in periodic metallic nanoslit array systems [37]. The interaction between cavity modes and surface modes leads to the formation of a plasmonic band gap, with suppressed transmission inside the gap, and enhanced transmission at the other band edge. However, the capped nanoslits had a sharper Fano resonance due to a higher reflection at the top interface, and efficient coupling between the cavity and SPR mode [12].

### 3.2. Wavelength Sensitivity and Intensity Sensitivity of the Capped Metallic Nanoslits

To determine the sensitivities of the capped aluminum nanoslits, we measured the Fano spectra under different surface refractive index conditions. The refractive index was controlled by injecting purified water mixed with various ratios of glycerin onto the sample surface. The nanostructure had a period of 470 nm. Figure 3d shows the transmission spectra of the capped aluminum nanoslits with various water/glycerin mixtures for normally-incident TM-polarized light. The nanostructure was fabricated using the rapid hot-embossing method. The structure parameters were H = 35 nm, T = 42 nm, W = 60 nm, and P = 470 nm. There were sharp Fano resonances in the spectra. When surface refractive index increased, the Fano resonance was red-shifted. Figure 3e shows the Fano resonant wavelength as a function of surface refractive index. The slope of the fitting curve shows that the wavelength sensitivity was 467 nm/RIU for the resonance peak. The sensitivity is close to the theoretical sensitivity in Equation (3), which indicates that the *S_λ_* is close to the period of the nanostructures. The wavelength sensitivity is comparable with most metallic nanostructure-based sensors using SPR or localized surface plasmon resonance (LSPR). It is noted that the Fano resonance had a very sharp resonant slope, therefore the intensity sensitivity (intensity change at a fixed wavelength under refractive index change) will be much higher than conventional SPR sensors. Figure 3f shows the normalized intensity change against the refractive index. The slope of the fitting curve shows that the intensity sensitivity was 29,345%/RIU. This measured intensity sensitivity is much higher than the reported intensity sensitivities of gold nanoslit, nanohole or nanogrid arrays: ~1000%/RIU–10,000%/RIU [18,22,38], and prism-based SPR sensors: ~15,000%/RIU [2]. In our measurement, using a simple while-light source and a cheap USB-based mini-spectrometer, the intensity noise was 0.7%. The refractive index resolution reached 2.38 × 10^−5^ RIU. The light source and spectrometer can be further improved to have a lower noise, 0.2%. The capped aluminum nanoslits can achieve a resolution of 6.8 × 10^−6^ RIU, which is comparable with commercial prism-based SPR machines using a complicated high-resolution angular detection method. To compare the sensitivity with previous works, we calculated the figure of merit (FOM = *S_λ_*/bandwidth) which is proportional to the intensity sensitivity. The measured bandwidth of the Fano resonant peak was 3.1 nm, and the wavelength sensitivity was 467 nm/RIU. Thus, the FOM value was 150 in the visible light region. The obtained FOM is higher than that of the previously reported FOMs in nanostructure-based aluminum sensors [31,39,40,41,42]. It was noted that the current geometric parameter is not the most sensitive structure. In our previous work, we have studied the effect of geometric parameters of the capped aluminum nanoslits on the surface sensitivity. For biolayer detections, the maximum surface sensitivity occurred at the dip of asymmetric Fano profile. The optimal Fano factor was close to −1.3 [43].

**Figure 4:**
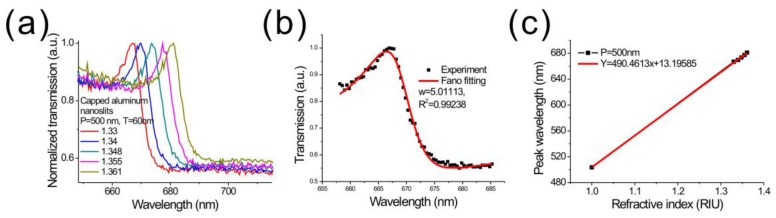
(**a**) The transmission spectra of the capped aluminum nanoslits with various water/glycerin mixtures. The sample was made by using dielectric-heating method; (**b**) the Breit–Wigner–Fano fitting of the Fano resonance; (**c**) the peak wavelength shift against the refractive index of medium for the nanostructure. The wavelength sensitivity was 490.46 nm/RIU.

Figure 4a shows the transmission spectra under different refractive index media for a normally-incident TM-polarized light. The nanostructures were made using the dielectric-heating method on a PETG film. The structure was a capped aluminum nanoslit array with a period of 500 nm. When surface refractive index increased, the Fano resonance was red-shifted. Figure 4b shows the Breit–Wigner–Fano fitting result. According to the fitting equation, for the Fano resonance at the air/aluminum interface, the linewidth (*w*) and Fano factor (*q*) were 5 nm and −1.84, respectively. The width was a little higher compared to the rapid hot-embossing method. Figure 4c shows the resonant wavelength as a function of surface refractive index. The slope of the fitting curve shows that the wavelength sensitivity was 490.46 nm/RIU for the resonance peak, which was similar to the performance of nanostructures made by the hot-embossing method. According to the wavelength sensitivity and linewidth, the FOM value was 98.1 in the visible light region. It is noted that both hot-embossing and dielectric-heating methods take less than 10 s for making nanostructures. The Ni–Co mold of the hot-embossing method needs to be kept at above 150 °C, while the dielectric-heating method is only active during the imprinting period. The dielectric-heating consumes less electric power than the hot-embossing method. However, the hot-embossing method uses a higher temperature and pressure during the imprinting process, resulting in a deeper nanostructure. Therefore, the BW–SPP resonance is stronger and the FOM is better than that of the dielectric-heating method.

### 3.3. Bio-Interaction Measurements Using Fano Resonances in Capped Aluminum Nanoslits

**Figure 5:**
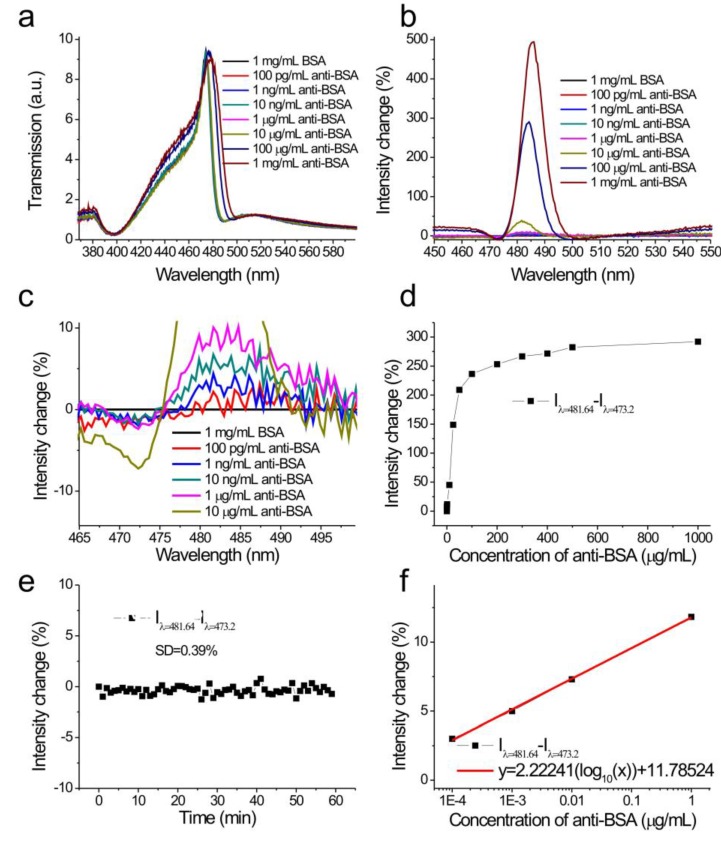
(**a**) The measured transmission spectra in 1 mg/mL bovine serum albumin (BSA) and different concentrations of anti-BSA solutions from 100 pg/mL to 1 mg/mL; (**b**) the spectral intensity changes caused by different concentrations of anti-BSA solutions. The transmission spectrum of the BSA solution was set as a reference; (**c**) the enlarged spectral intensity changes caused by different concentrations of anti-BSA solutions; (**d**) the intensity change difference (I_diff_ = ∆I/I_λ = 481.64_−∆I/I_λ = 473.20_) as a function of the concentration of anti-BSA; (**e**) the intensity change as a function of the logarithm of the logarithm of the concentration of the anti-BSA solution; (**f**) the intensity change (I_λ = 481_−I_λ = 473_) as a function of time.

The capped aluminum nanoslit array using the hot-embossing method was applied to study the antigen and antibody interactions, for measuring the limit of detection (LOD) of biosamples. Both wavelength and intensity interrogation methods were compared. Figure 5a shows the measured transmission spectra in 1 mg/mL BSA and different concentrations of anti-BSA solutions, from 100 pg/mL to 1 mg/mL, for normally incident TM-polarized light. The Fano resonances were red-shifted as the concentration increased and the transmitted intensity changed. We analyzed the spectra and set the transmission spectra of the BSA solution as references. Obviously, the detectable concentration with wavelength interrogation was 1 μg/mL, which was limited by the wavelength resolution (0.4 nm) of the spectrometer. Figure 5b shows the spectral intensity changes caused by different concentrations of anti-BSA solutions. Obviously, even for a concentration of 100 pg/mL, the intensity had a small change at a wavelength of 481 nm as shown in the inset of Figure 5b. Since the change is negative below the resonance wavelength and positive above the resonance wavelength, we used the intensity difference (I_diff_) above and below the resonance wavelength to evaluate the intensity sensitivity. In the calculations, I_diff_ is defined as the difference between the intensity changes at 481.64 nm and 473.2 nm. Figure 5c shows the intensity difference, I_diff_ = ∆I/I (*λ* = 481.64 nm)–∆I/I (*λ* = 473.20 nm), as a function of the anti-BSA concentration. The intensity changes at these two wavelengths increased and decreased, with the increase of the anti-BSA concentration, respectively. Therefore, the intensity difference, I_diff_, can reduce disturbances such as light source fluctuation. The intensity difference increased, and then gradually became saturated as the concentration increased. The responses were 3, 5, 7.8, 11.8, 45.1, 236.3, and 292.0% for 0.0001, 0.001, 0.01, 1, 10, 100, and 1000 μg/mL, respectively. There was a linear correlation between the response and the logarithm of the concentration when the concentration was less than 1 μg/mL, as shown in Figure 5d. The calibration curve was described by y = 2.22241(log_10_(x)) + 11.78524, R^2^ = 0.99893. In addition, the measured intensity noise, extracted from the inset in Figure 5d, was 0.39% (one standard deviation of the response). Based on the calibration curve and measurement system noise (1.17%, 3 standard deviations of the response), the LOD of the surface concentration (detectable concentration) of anti-BSA can be obtained by a linear regression equation. This yields a theoretical detection limit of 16.7 pg/mL.

## 4. Conclusions

**Figure 6:**
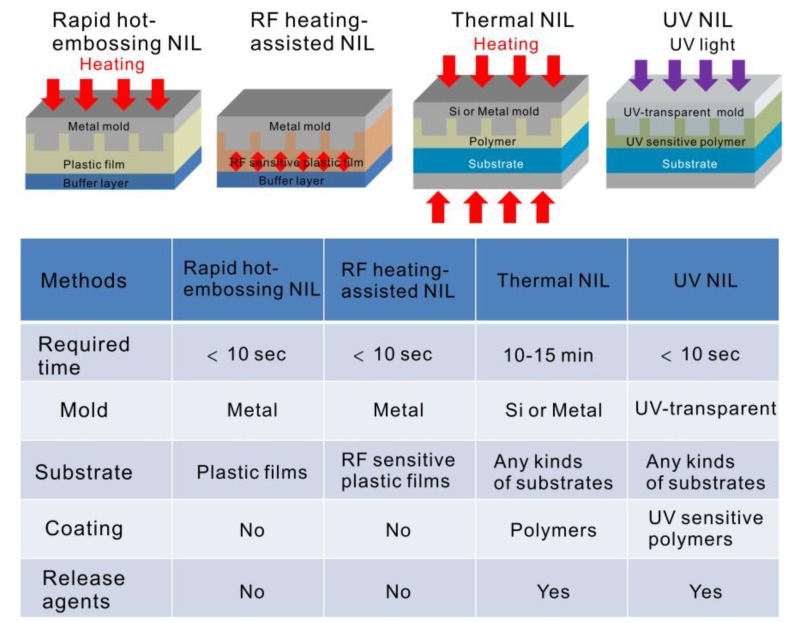
Comparison of various nanoimprinting methods.

We proposed two approaches—rapid hot-embossing and dielectric-heating nanoimprinting—for low-cost, rapid and high-throughput fabrication of nanostructures for SPR-based biosensors. Each imprinting process was completed within several seconds, and release agents were not utilized. The low-cost, large-area, and highly sensitive aluminum nanostructures on A4 size plastic films were fabricated by utilizing the rapid hot-embossing method combined with evaporation of metallic film. The narrowest bandwidth of the Fano resonance was only 2.7 nm in the visible light region. The nanostructure reaches a figure of merit of 150 and an intensity sensitivity up to 29,345%/RIU. Besides, the protein–protein interaction experiments verified the high sensitivity of the structures, and a theoretical detection limit of 16.7 pg/mL anti-BSA can be achieved. It is worthwhile to compare both techniques with conventional nano-imprinting lithography (NIL). Figure 6 shows this comparison. As compared to thermal NIL, it often takes several minutes to complete the imprinting process. The UV-NIL method can make nanostructures within a short time. However, it needs UV curable resins coated onto a substrate, and UV-transparent patterned templates. Besides, release agents for demolding are frequently required for UV-NIL and thermal NIL methods. In our methods, the rapid hot-embossing method only heats the metal mold, and RF power primarily heats the RF-sensitive plastics. Both methods take advantage of the quick surface heating and cooling. No release agents for demolding are required. In addition, a thick plastic film can be placed below the substrate and acts as a pressure buffer layer. With this buffer layer, the embossing pressure is uniformly distributed on the sample surface. It benefits the fabrication of uniform and large-area nanostructures. As the nanostructures were fabricated on the plastic film, it can be directly integrated to the microfluidic devices made on the plastic film using hot-embossing nanoimprint lithography [44]. The sample treatment and plasmonic multiplexed detection can be conducted on a chip. These low-cost and high-throughput fabrication techniques can benefit biosensing and other applications.

## References

[1] Raether H. (1988). Surface plasmons on smooth and rough surfaces and on gratings. Springer Tracts in Modern Physics.

[2] Homola J., Yee S.S., Gauglitz G. (1999). Surface plasmon resonance sensors. Sens. Actuator B Chem..

[3] Maier S.A. (2007). Plasmonics: Fundamentals and Applications.

[4] Homola J. (2008). Surface plasmon resonance sensors for detection of chemical and biological species. Chem. Rev..

[5] Anker J.N., Hall W.P., Lyandres O., Shah N.C., Zhao J., Van Duyne R.P. (2008). Biosensing with plasmonic nanosensors. Nat. Mater..

[6] Brolo A.G., Gordon R., Leathem B., Kavanagh K. L. (2004). Surface plasmon sensor based on the enhanced light transmission through arrays of nanoholes in gold films. Langmuir.

[7] Henzie J., Lee M.H., Odom T.W. (2007). Multiscale patterning of plasmonic metamaterials. Nat. Nanotechnol..

[8] Gordon R., Sinton D., Kavanagh K.L., Brolo A.G. (2008). A new generation of sensors based on extraordinary optical transmission. Acc. Chem. Res..

[9] Yanik A.A., Cetin A.E., Huang M., Artar A., Mousavi S.H., Khanikaev A., Connor J.H., Shvets G., Altug H. (2011). Seeing protein monolayers with naked eye through plasmonic Fano resonances. Proc. Natl. Acad. Sci. USA.

[10] Lee K.L., Lee C.W., Wang W.S., Wei P.K. (2007). Sensitive biosensor array by using surface plasmon resonance on metallic nanoslits. J. Biomed. Opt..

[11] Shen Y., Zhou J., Liu T., Tao Y., Jiang R., Liu M., Xiao G., Zhu J., Zhou Z.K., Wang X. (2013). Plasmonic gold mushroom arrays with refractive index sensing figures of merit approaching the theoretical limit. Nat. Commun..

[12] Lee K.L., Huang J.B., Chang J.W., Wu S.H., Wei P.K. (2015). Ultrasensitive biosensors using enhanced Fano resonances in capped gold nanoslit arrays. Sci. Rep..

[13] Lee K.L., Chang C.C., You M.L., Pan M.Y., Wei P.K. (2016). Enhancing the surface sensitivity of metallic nanostructures using oblique-angle-induced Fano resonances. Sci. Rep..

[14] Lesuffleur A., Im H., Lindquist N.C., Lim K.S., Oh S.H. (2008). Laser-illuminated nanohole arrays for multiplex plasmonic microarray sensing. Opt. Express.

[15] Stewart M.E., Mack N.H., Malyarchuk V., Soares J.A., Lee T.W., Gray S.K., Nuzzo R.G., Rogers J.A. (2006). Quantitative multispectral biosensing and 1D imaging using quasi-3D plasmonic crystals. Proc. Natl. Acad. Sci. USA.

[16] Das M., Hohertz D., Nirwan R., Brolo A.G., Kavanagh K.L., Gordon R. (2011). Improved Performance of Nanohole Surface Plasmon Resonance Sensors by the Integrated Response Method. IEEE Photon. J..

[17] Lee K.L., Wei P.K. (2010). Enhancing surface plasmon detection using ultrasmallnanoslits and multispectral integration method. Small.

[18] Lee K.L., Chih M.J., Shi X., Ueno K., Misawa H., Wei P.K. (2012). Improving surface plasmon detection in gold nanostructures using a multi-polarization spectral integration method. Adv. Mater..

[19] Lee S.H., Johnson T.W., Lindquist N.C., Im H., Norris D.J., Oh S.H. (2012). Linewidth-Optimized Extraordinary Optical Transmission in Water with Template-Stripped Metallic Nanohole Arrays. Adv. Funct. Mater..

[20] Nagpal P., Lindquist N.C., Oh S.H., Norris D.J. (2009). Ultrasmooth patterned metals for plasmonics and metamaterials. Science.

[21] Hegner M., Wagner P., Semenza G. (1993). Ultralarge atomically flat template-stripped Au surfaces for scanning probe microscopy. Surf. Sci..

[22] Lee K.L., Chen P.W., Wu S.H., Huang J.B., Yang S.Y., Wei P.K. (2012). Enhancing surface plasmon detection using template-stripped gold nanoslit arrays on plastic film. ACS Nano.

[23] Fano U. (1941). The theory of anomalous diffraction gratings and of quasi-stationary waves on metallic surfaces (Sommerfeld’s waves). J. Opt. Soc. Am..

[24] Miroshnichenko A.E., Flach S., Kivshar Y.S. (2010). Fano resonances in nanoscale structures. Rev. Mod. Phys..

[25] Luk’yanchuk B., Zheludev N.I., Maier S.A., Halas N.J., Nordlander P., Giessen H., Chong C.T. (2010). The Fano resonance in plasmonic nanostructures and metamaterials. Nat. Mater..

[26] Gao H., Yang J.C., Lin J.Y., Stuparu A.D., Lee M.H., Mrksich M., Odom T.W. (2010). Using the angle-dependent resonances of molded plasmonic crystals to improve the sensitivities of biosensors. Nano Lett..

[27] Tsai W.S., Lee K.L., Pan M.Y., Wei P.K. (2013). Increased detection sensitivity of surface plasmon sensors using oblique induced resonant coupling. Opt. Lett..

[28] Lahav A., Auslender M.I., Abdulhalim I. (2008). Sensitivity enhancement of guided-wave surface-plasmon resonance sensors. Opt. Lett..

[29] Liu N., Weiss T., Mesch M., Langguth L., Eigenthaler U., Hirscher M., Sonnichsen C., Giessen H. (2010). Planar metamaterial analogue of electromagnetically induced transparency for plasmonic sensing. Nano Lett..

[30] Menezes J.W., Ferreira J., Santos M.J. L., Cescato L., Brolo A.G. (2010). Large-area fabrication of periodic arrays of nanoholes in metal films and their application in biosensing and plasmonic-enhanced photovoltaics. Adv. Funct. Mater..

[31] Skinner J.L., Hunter L.L., Talin A.A., Provine J., Horsley D.A. (2008). Large-area subwavelength aperture arrays fabricated using nanoimprint lithography. IEEE Trans. Nanotechnol..

[32] Lee S.H., Bantz K.C., Lindquist N.C., Oh S.H., Haynes C.L. (2009). Self-assembled plasmonicnanohole arrays. Langmuir.

[33] Aksu S., Huang M., Artar A., Yanik A.A., Selvarasah S., Dokmeci M.R., Altug H. (2011). Flexible plasmonics on unconventional and nonplanar substrates. Adv. Mater..

[34] Im H., Lee S.H., Wittenberg J.N., Johnson T.W., Lindquist C.N., Nagpal P., Norris D.J., Oh S.H. (2011). Template-Stripped Smooth Ag Nanohole Arrays with Silica Shells for Surface Plasmon Resonance Biosensing. ACS Nano.

[35] Lindquist N.C., Johnson T.W., Norris D.J., Oh S.H. (2011). Monolithic Integration of Continuously Tunable Plasmonic Nanostructures. Nano Lett..

[36] Gordon R. (2006). Light in a subwavelength slit in a metal: propagation and reflection. Phys. Rev. B.

[37] De Ceglia D., Vincenti M.A., Scalora M., Akozbek N., Bloemer M.J. (2011). Plasmonic band edge effects on the transmission properties of metal gratings. AIP Adv..

[38] Yang J.C., Ji J., Hogle J.M., Larson D.N. (2008). Metallic nanohole arrays on fluoropolymer substrates as small label-free real-time bioprobes. Nano Lett..

[39] Norek M., Włodarski M., Matysik P. (2014). UV plasmonic-based sensing properties of aluminum nanoconcave arrays. Curr. Appl. Phys..

[40] Canalejas-Tejero V., Herranz S., Bellingham A., Moreno-Bondi M.C., Barrios C.A. (2014). Passivated aluminum nanohole arrays for label-free biosensing applications. ACS Appl. Mater. Inter..

[41] King N.S., Liu L., Yang X., Cerjan B., Everitt H.O., Nordlander P., Halas N.J. (2015). Fano resonant aluminum nanoclusters for plasmonic colorimetric sensing. ACS Nano.

[42] Ahmadivand A., Golmohammadi S., Pala N. (2015). Fano resonances in plasmonic aluminum nanoparticle clusters for precise gas detection: Ultra-sensitivity to the minor environmental refractive index perturbations. Photon. Nanostruct. Fundam. Appl..

[43] Lee K.L., Hsu H.Y., You M.L., Chang C.C., Pan M.Y., Shi X., Ueno K., Misawa H., Wei P.K. (2017). Highly Sensitive Aluminum-Based Biosensors using Tailorable Fano Resonances in Capped Nanostructures. Sci. Rep..

[44] Malic L., Morton K., Clime L., Veres T. (2013). All-thermoplastic nanoplasmonic microfluidic device for transmission SPR biosensing. Lap Chip.

